# Antimalarials may reduce cancer risk in patients with systemic lupus erythematosus: a systematic review and meta-analysis of prospective studies

**DOI:** 10.1080/07853890.2021.1981547

**Published:** 2021-09-23

**Authors:** Xian-Bao Li, Nv-Wei Cao, Xiu-Jie Chu, Hao-Yue Zhou, Hua Wang, Si-Jie Yu, Dong-Qing Ye, Bao-Zhu Li

**Affiliations:** a Department of Epidemiology and Biostatistics, School of Public Health, Anhui Medical University, Hefei, Anhui, China; b Anhui Provincial Laboratory of Inflammatory and Immune Diseases, Hefei, Anhui, China; c Department of Clinical Medicine “5 + 3” Integration, Second Clinical Medical College, Anhui Medical University, Hefei, Anhui, China

**Keywords:** Systemic lupus erythematosus, cancer, antimalarials, hydroxychloroquine

## Abstract

**Objective:**

To investigate the effect of antimalarials on cancer risk in patients with systemic lupus erythematosus (SLE).

**Methods:**

PubMed, EMBASE, Web of Science, and the Cochrane Library were searched from their inception to October 3, 2020. Relative risk (RR) with 95% confidence intervals (CI) was used to evaluate the results. Subgroup analyses were used to assess heterogeneity. A funnel plot was used to explore publication bias. STATA was applied for all analyses.

**Results:**

A total of nine studies consisted of four nested case–control, two case–cohort and three cohort studies were included. The results showed that antimalarials might reduce the risk of cancer in SLE (RR = 0.68, 95%CI: 0.55–0.85). In the subgroup analysis of four nested case–control and two case–cohort studies, the pooled RR was estimated as 0.69 (95% CI: 0.60–0.80). In four studies about hydroxychloroquine, the pooled RR was estimated as 0.70 (95% CI: 0.53–0.93). Antimalarials might reduce the risk of cancer in SLE among the Asian population (RR = 0.66; 95% CI: 0.49–0.88) (*I*^2^ = 43.1%, *p* = .173). And the consistent result was also found in SLE from multiple centres (RR = 0.72; 95%CI: 0.60–0.87) (*I*^2^ = 0%, *p* = .671). On disease course- and comorbidities-matched studies, the pooled RRs were 0.69 (95% CI: 0.52–0.93) and 0.59 (95% CI: 0.46–0.75), respectively.

**Conclusion:**

Results of this meta-analysis showed that antimalarial drugs might be protective factors for cancer in SLE. Hydroxychloroquine might be a protective factor for cancer in SLE patients.

**KEY MESSAGES**
- Antimalarials might be protective factors for cancer in SLE.
- Hydroxychloroquine might be a protective factor for cancer in SLE patients.
- The first article to perform the meta-analysis of antimalarial drugs on the risk of cancer in SLE patients.

## Introduction

Systemic lupus erythematosus (SLE) is an autoimmune disease that often occurs in women and affects multiple organs [1,2]. Patients with SLE have a higher mortality rate than the general population [3,4]. The main causes of death in SLE patients are infection, cardiovascular disease, and cancer [5,6]. Compared to the general population, previous studies have shown a higher incidence of cancer in SLE patients, such as lymphoma, vulva cancer, lung cancer, thyroid cancer, cervical cancer, and kidney cancer [7–11].

At present, corticosteroids, hydroxychloroquine (HCQ), and immunosuppressants are most commonly used in the clinical treatment of SLE. Immunoregulatory and anti-inflammatory effects of antimalarials have beneficial effects on the outcomes of SLE patients. It might improve the survival and remission rates [12], ameliorate disease activity [13], reduce accrual of new disease-related damage [14,15], and decrease infection rates [16]. HCQ and chloroquine (CQ) are autophagy inhibitors that inhibit autophagy by affecting lysosomes [17]. Current studies have found that autophagy can regulate cell cycle progression, thus playing an important role in cancer [18–20].

Some studies have reported relationships between antimalarials and cancer in SLE patients, but the results were controversial. Ruiz-Irastorza G put forward a hypothesis that antimalarial drugs might have a protective effect on cancer in SLE patients [21]. But Dey found that the risk of tumours in SLE patients was not related to drug, dose, or disease duration [22]. Therefore, a meta-analysis is needed to explore whether taking antimalarials is associated with the risk of cancer in SLE patients.

## Methods

### Data sources and searches

This review was conducted by PRISMA guidelines [23] (Supplementary Material 1). We systematically searched EMBASE, Web of Science, PubMed, and the Cochrane Library from their inception to the end of October 3, 2020. The search strategy included the MeSH and text words as ((lupus erythematosus, systemic [MeSH Terms] OR systemic lupus erythematosus OR lupus OR SLE) AND (neoplasms [MeSH Terms] OR carcinoma [MeSH Terms] OR cysts [MeSH Terms] cancer OR carcinoma OR malignancy OR neoplasm OR neoplasia OR tumour)) AND (antimalarials [MeSH Terms] OR hydroxychloroquine [MeSH Terms] OR chloroquine [MeSH Terms] OR antimalarial agents OR antimalarial drugs OR anti-malarials OR antimalarial OR hydroxychloroquine OR HCQ OR chloroquine). In addition, relevant articles outside the search list were manually searched.

### Study selection

Studies were eligible if they met the following criteria: (1) All included patients met the American College of Rheumatology criteria or international classification of disease criteria for the diagnosis of SLE. (2) The study provided the cancer outcomes in SLE patients who took antimalarials (antimalarials+) and controls who did not take antimalarials (antimalarials−). (3) The study was designed by cohort, case–cohort, or nested case–control. The study would be excluded if met following contents: (1) The outcome was cancer with precancerous lesions. (2) The included patients had cutaneous lupus erythematosus.

### Data extraction and quality assessment

The relevant information, including first author, research period, year of publication, type of study, study population, disease course, SLE and cancer diagnostic criteria, the number of SLE patients taking antimalarials, and the number of people who developed cancer in each group, were extracted independently by two reviewers (XBL and NWC). In these studies, disease duration was defined as the duration from the date of SLE diagnosis to that of their cancer diagnosis [22]. When these reviewers had inconsistent opinions on an article, disagreements were resolved through discussions with another reviewer (XJC). Two reviewers (XBL and NWC) independently evaluated the risk of potential bias by the Newcastle–Ottawa Scale (NOS). Disagreements were resolved through discussions with another reviewer (XJC).

### Data synthesis and analysis

Excel 2019 and STATA 11.0 (Stata Corp. LP, College Station, TX, USA) were used for data extraction and meta-analysis. Data was expressed by relative risk (RR) with its associated 95% confidence intervals (CI). Inverse variance with random effects models was used for data analysis. Inconsistency (*I*^2^) was calculated to determine heterogeneity. *I*^2^ < 50% indicated that the heterogeneity of included studies was acceptable. Factors, such as research type, region, types of antimalarials, gender, age, disease course, and comorbidities may induce heterogeneity. If included studies reported information about these factors, subgroup analyses were conducted to explore sources of heterogeneity. Sensitivity analysis was used to assess the robustness of estimates. Funnel plots, Egger’s test, and Begg’s test were applied to evaluate publication bias. *p* < .05 was defined as statistically significant.

## Results

### Study selection

**Figure 1.**
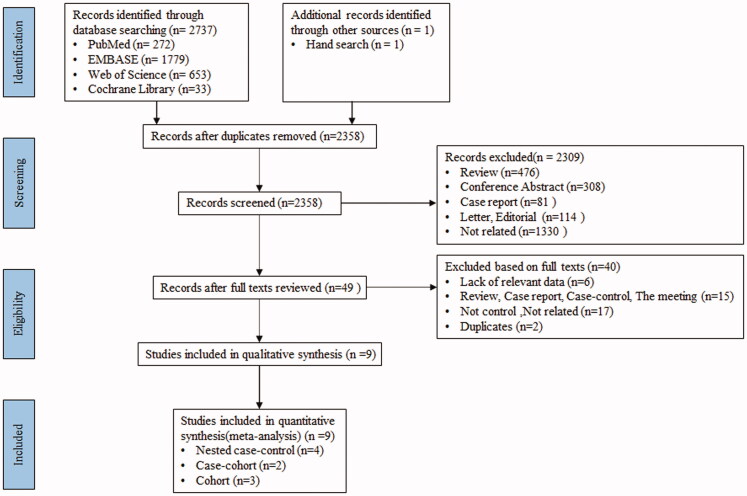
Flow diagram of the literature search and study inclusion.

A total of 2737 articles were searched at first from four databases. Two thousand six hundred and eighty-nine articles were excluded after deleting duplication and screening titles and abstracts. After reviewing the full text of the remaining, 40 studies were excluded (lack of relevant data, *n* = 6; review, case report, the meeting, case–control, *n* = 15; not control, *n* = 1; not related, *n* = 16; duplicates, *n* = 2), and eight studies were finally included [21,24–30]. One study was also included after manually searching [22]. In the end, a total of nine articles were included in this meta-analysis (Figure 1).

### Study characteristics

The characteristics of these included studies were detailed in Table 1. Three cohorts, four nested case–control, and two case–cohort studies were included. Four studies showed the effects of HCQ on cancer in SLE, while five did not state specific types of antimalarials. Three included studies were conducted in Asia populations, three were in European populations, and the rest were in multiple populations. Among included studies, four matched the age and gender of case and control groups [22,25,29,30], three matched the course of disease in two groups [22,25,28], and two matched comorbidities of case and control groups [25,29]. Other detailed information was shown in Supplemental Table 1.

**Table 1.** Characteristics of included studies.

Author, Year	Drug	Design	Region	SLE diagnostic criteria	Cancer diagnostic criteria	Cancer/ control female	Cancer/ control age at SLE diagnosis	Cancer/control disease course of SLE	Matched characteristics	No. of cancer in use drug/ No. of all in use drug	No. of cancer in control/ No. of all in control	Study period
Guo JY 2020	HCQ	Nested case–control	Asia	ACR1997	Histological analyses	49/192	41/33	60/60 months	Age, gender, complications, disease course	29/184	22/71	2010–2019
Cai SZ 2020	HCQ	Nested case–control	Asia	ICD-9 710.0	ICD-9 140-208	NA	NA	NA	Age, gender	20/39	23/40	1997–2013
Hsu CY 2017	HCQ	Nested case–control	Asia	ACR1997	Tissue proof	295/1180	44.8/44	NA	Age, gender, complications	289/1517	41/133	2001–2013
Dey D 2013	HCQ	Nested case–control	Europe	ACR1997	Histological or autopsy reports.	NA	NA	NA	Age, gender, disease course	24/91	9/36	1978–2010
Bernatsky S 2020	Antimalarial	Cohort	North America, Europe and Asia	ACR1997	Medical files including pathology reports	48/1432	45.6/34.2	5.5/5.6 months	NA	50/1313	15/355	1999–2011
Wadstrom H 2017	Antimalarial	Cohort	Europe	ICD-8 734.1, ICD-9 710.0, ICD-10 M32	Histology or cytology analyses	5/4971	NA	NA	NA	0/1942	5/3034	2006–2012
Bernatsky S 2014	Antimalarial	Case–cohort	North America, Europe and Asia	ACR1997	Regional registry OR medical chart	60/4511	45.2/38.5	6.2/6.2 years	NA	55/4077	20/959	NA
Bernatsky S 2008	Antimalarial	Case–cohort	North America, Europe and Asia	ACR1997 OR clinical criteria	Regional cancer registry linkages	221/489	42/35	1/1 years	Disease course	100/385	146/399	1958–2000
Ruiz-Irastorza, G 2007	Antimalarial	Cohort	Europe	ACR1997	Radiological and/or histological	142/67*	34/42^†^	NA	NA	2/156	11/79	1975–2005

HCQ: hydroxychloroquine; NA: not applicable; ACR: American College of Rheumatology criteria; ICD: International Classification of Diseases.

*No. of female taking antimalarials/No. of female not taking antimalarials.

^†^Mean age at diagnosis (drug-use/drug-free).

### Quality of included studies

All NOS scores ≥7 indicated the high quality of all included studies (Table 2). All groups in each study used the same survey methods. None of the controls had a history of disease in case–control studies, and none of the subjects developed the disease under study at the beginning of cohort studies. Cases were well-represented in case–control studies, and exposures were representative in cohort studies. The investigation of exposure and the determination of outcome had reliable sources.

**Table 2.** Methodological quality of studies included in the meta-analysis.

References	Cases definition	Representativeness of cases	Selection of controls	Definition of controls	Control for important factor or additional factors^a^	Ascertainment of exposure	Same method of ascertainment for participants	Non-responsive rate	Total quality scores
Nested case–control or case–cohort or case–control studies									
Guo JY [23]	★	–	–	★	★★	★	★	★	7
Cai, SZ [28]	★	★	★	★	★★	★	★	★	9
Hsu CY [27]	★	★	★	★	★★	★	★	★	9
Bernatsky S [25]	–	★	★	★	★	★	★	★	7
Bernatsky S [26]	–	★	★	★	★★	★	★	★	8
Dey D [29]	–	–	–	★	★★	★	★	–	7
	Representativeness of the exposed cohort	Selection of unexposed cohort	Assessment of exposure	Absence of outcome at the start of study		Outcome assessment	Follow-up period	Adequacy of follow-up	
Cohort studies									
Bernatsky, S [24]	★	★	★	★	–	★	★	★	7
Wadstrom H [21]	★	★	★	★	–	★	★	★	7
Ruiz-Irastorza, G [22]	–	★	★	★	★	★	★	★	7

^a^Studies that controlled for age and sex received 1 star. Studies that controlled for other risk factors received an additional star.

### Antimalarials reduce the risk of cancer in SLE

**Figure 2.**
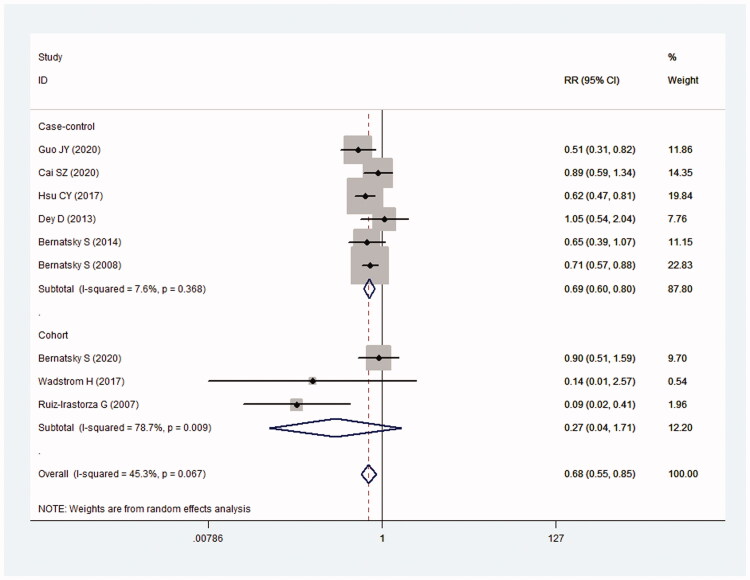
Relationship between the use of antimalarials and cancer risk in SLE.

Results showed that the risk of cancer in the antimalarials+ group was lower than that in the antimalarials− group (RR = 0.68; 95%CI: 0.55–0.85) (*I*^2^ = 45.3%, *p* = .067) (Figure 2). In four nested case–control and two case–cohort studies, the risk of cancer was lower in the antimalarials+ group than that in the antimalarials− group (RR = 0.69; 95%CI, 0.60–0.80) (*I*^2^ = 7.6%; *p* = .365) (Figure 2). However, in three cohort studies, there was no significant difference between the two groups (RR = 0.27; 95%CI: 0.04–1.71) (*I*^2^ = 78.7%; *p* = .009) (Figure 2).

### Subgroup analysis

**Figure 3.**
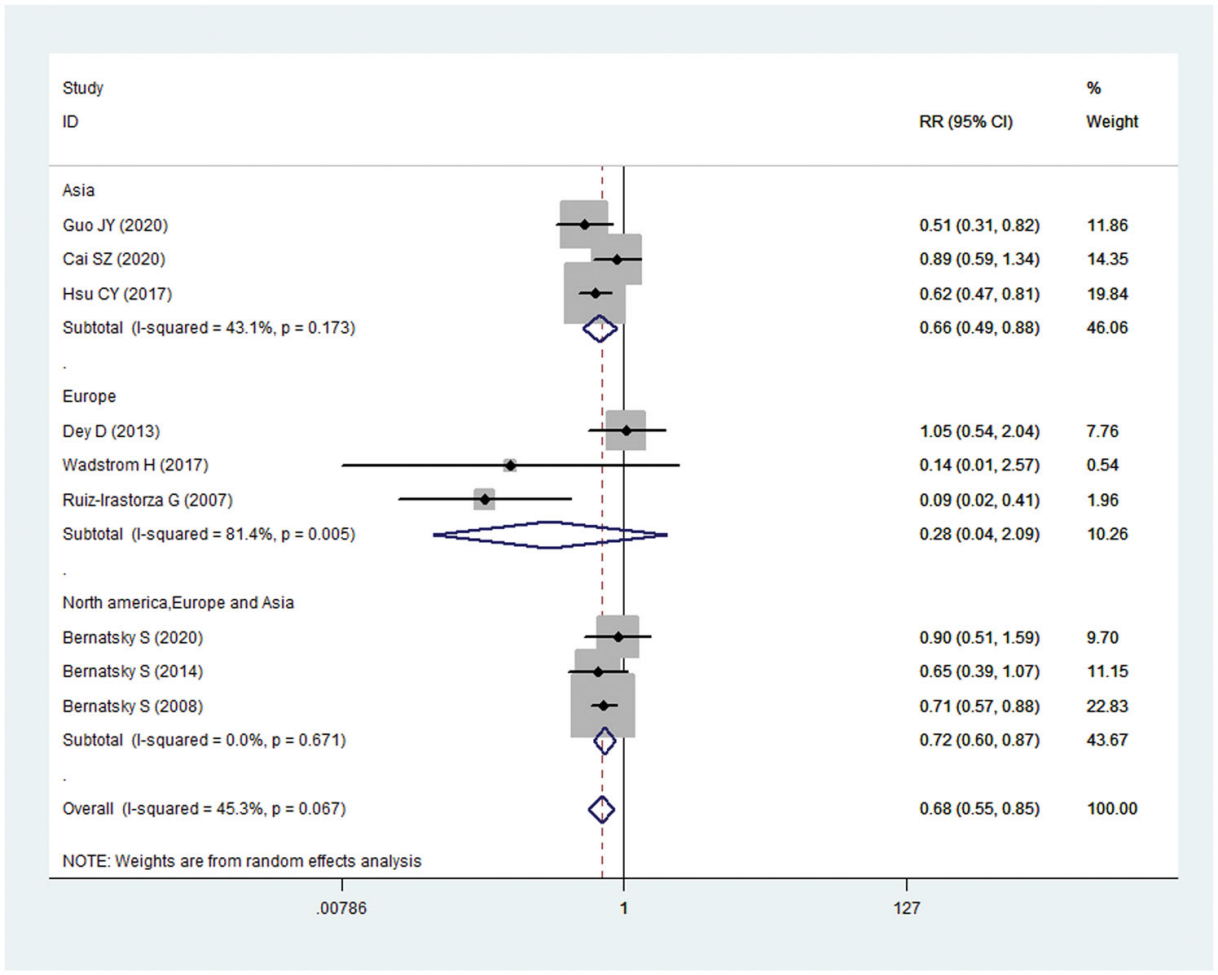
Relationship between the use of antimalarials and cancer risk in SLE in different regions.

Among the Asian population, the antimalarials+ group had a lower risk of cancer (RR = 0.66; 95%CI: 0.49–0.88) (*I*^2^ = 43.1%, *p* = .173). And a consistent result was also found in SLE from multiple centres (RR = 0.72; 95%CI: 0.60–0.87) (*I*^2^ = 0%, *p* = .671). Whereas no significant difference was found among the European population (RR = 0.28; 95%CI: 0.04–2.09) (*I*^2^ = 81.4%; *p* = .005) (Figure 3).

On studies matching disease course, it was found that the risk of cancer in the antimalarials+ group was lower than that in the antimalarials− group (RR = 0.69; 95%CI, 0.52–0.93) (*I*^2^ = 37.1%; *p* = .204) (Supplemental Figure 1(a)). On studies matching complications, the pooled RR was 0.59 (95% CI: 0.46–0.75) (*I*^2^ = 0%, *p* = .490) (Supplemental Figure 1(b)). On studies matching age and gender, the pooled RR was 0.70 (95%CI, 0.53–0.93) (*I*^2^ = 43.1%; *p* = .153) (Supplemental Figure 1(c)).

**Figure 4.**
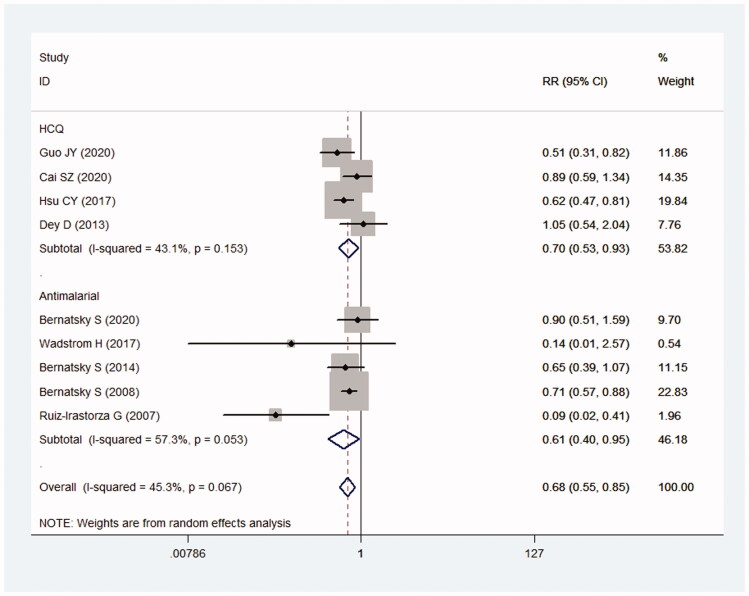
Relationship between the use of HCQ and cancer risk in SLE.

At present, HCQ is the most common antimalarial drug used to treat SLE. Results showed that HCQ reduced the risk of cancer in SLE patients (RR = 0.70; 95% CI: 0.53–0.93) (*I*^2^ = 43.1%, *p* = .153) (Figure 4). In five studies that did not state specific types of antimalarials, the result showed the pooled RR was 0.61 (95% CI: 0.40–0.95) (*I*^2^ = 57.3%, *p* = .053) (Figure 4).

### Funnel figure and sensitivity analysis

Results of Egger’s test (*t* = −0.88) and Begg’s test (*Z* = 0.52) indicated no significant publication bias (Supplemental Figure 2). Removing one article each time, overall RRs were around 0.68 (Supplemental Figure 3). It indicated that the results of this study were robust and not affected by any single study.

## Discussion

To the best of our knowledge, this is the first meta-analysis examining the risk of cancer in SLE patients taking antimalarials. Antimalarials might reduce cancer risk in SLE patients. In particular, HCQ might reduce the risk of cancer in SLE patients. In four nested case–control and two case–cohort, SLE patients in the antimalarials+ group had a lower risk of cancer than those in antimalarials− group. But the difference was not found in subgroup analysis in cohort studies.

Three phase I trials of HCQ combined with vorinostat, bortezomib and temsirolimus in the treatment of cancer showed that the combination therapy had anti-tumour activity and had potential effects on the treatment of tumours [31–33]. In a meta-analysis of seven clinical trials, autophagy-inhibitor-based therapy (HCQ or chloroquine combination therapy) had a better response in cancer treatment than chemotherapy or radiation without inhibiting autophagy [18]. Various published studies reported the association between the risk of cancer and antimalarials in patients with SLE. In a previous study with 14 cohorts, a reduced risk (adjusted hazard ratios = 0.55) was observed for lung cancer in SLE patients with cumulative use of antimalarial drugs over 5 years but did not reach statistical significance [34]. Feldman CH found a trend that people receiving HCQ had a lower rate of cervical dysplasia and cervical cancer compared with those who receive immunosuppressive drugs [35]. In our large-sample and multi-center meta-analysis, it was also indicated that antimalarials might have a protective effect on the risk of cancer in SLE.

The antitumor properties of antimalarials may be related to their promotion of macrophage transformation, inhibition of autophagy, and promotion of apoptosis [36]. HCQ and CQ are weak binary bases. By increasing the pH of lysosomes and other intracellular compartments, these drugs interfered with the function of phagocytosis and antigen presentation to T cells [37,38]. The increase of pH value in lysosomes would promote the transformation of tumour-associated macrophages (TAMS) from M2 phenotype to M1 phenotype [39]. M2-TAM can block immune monitoring and increase tumour progression and metastasis, while M1-TAM can release nitrogen oxide and interferon-γ to kill tumours [40]. Chloroquine exerts an anti-tumour effect by transforming tumour-promoting M2-TAM into tumour-suppressing M1-TAM. Current studies showed that autophagy played a different role in different stages of cancer [41]. Initially, inhibiting autophagy may encourage healthy cells to develop cancer [42]. But, in later stages of cancer, autophagy can enhance tumour progression and metastasis, and enhance the ability to respond to adverse microenvironmental conditions, such as hypoxia and nutritional deficiencies [43,44]. Inhibition of autophagy may increase environmental or treatment-induced stress to promote cancer cell death [45,46]. One reason for the survival of cancer cells is the ability to escape apoptosis. Beclin-2 protein can block mitochondrial apoptosis [47]. Antimalarial drugs promote mitochondrial apoptosis through block the apoptosis regulator (Beclin-2 protein), thereby promoting the apoptosis of cancer cells [48].

Several advantages were in this study. First, the data of our study was retrieved from prospective studies, including cohort studies, nested case–control studies, and case–cohort studies. The collection of exposure data in prospective studies is obtained by the investigator personally, and there is generally no recall bias, so the data is reliable. In prospective studies, because the exposure occurs before the occurrence of the disease and the causal time sequence is clear, the ability to test the aetiological hypothesis is stronger and the results are more reliable. Second, a large sample size of 14810 was included in this meta-analysis, indicating the high credibility of the results. Third, with all NOS scores ≥7, the qualities of studies included were relatively high. It demonstrated the high reliability and validity of the results.

However, our study has several limitations related to the quality of data in original sources. First, we were unable to fully assess the risk of bias in certain situations because the published studies did not provide enough details. Due to the lack of necessary data on malignant tumour types and drug dosages, our results may be underestimated. Therefore, the drug dosage and the drug situation for each cancer should be reported in further studies. Second, among different research types, the results of subgroups were inconsistent. Therefore, the results should be further verified in the future. Third, our data in this study came from observational studies, and we need to be cautious when interpreting the evidence of observational studies, because the results of observational studies are more biased than the results of randomized controlled trials. Therefore, more experiments are needed to prove this conclusion in the future.

## Conclusion

The result of this meta-analysis showed that antimalarials might be protective factors for cancer in SLE. HCQ might be a protective factor for cancer in SLE patients.

## Supplementary Material

Supplemental Material
