# Supplementary figures and images for: Antimalarials may reduce cancer risk in patients with systemic lupus erythematosus: a systematic review and meta-analysis of prospective studies
Source: Ann Med. 2021 Sep 23;53(1):1688–96. doi: 10.1080/07853890.2021.1981547 (PMC8462850; doi:10.1080/07853890.2021.1981547)

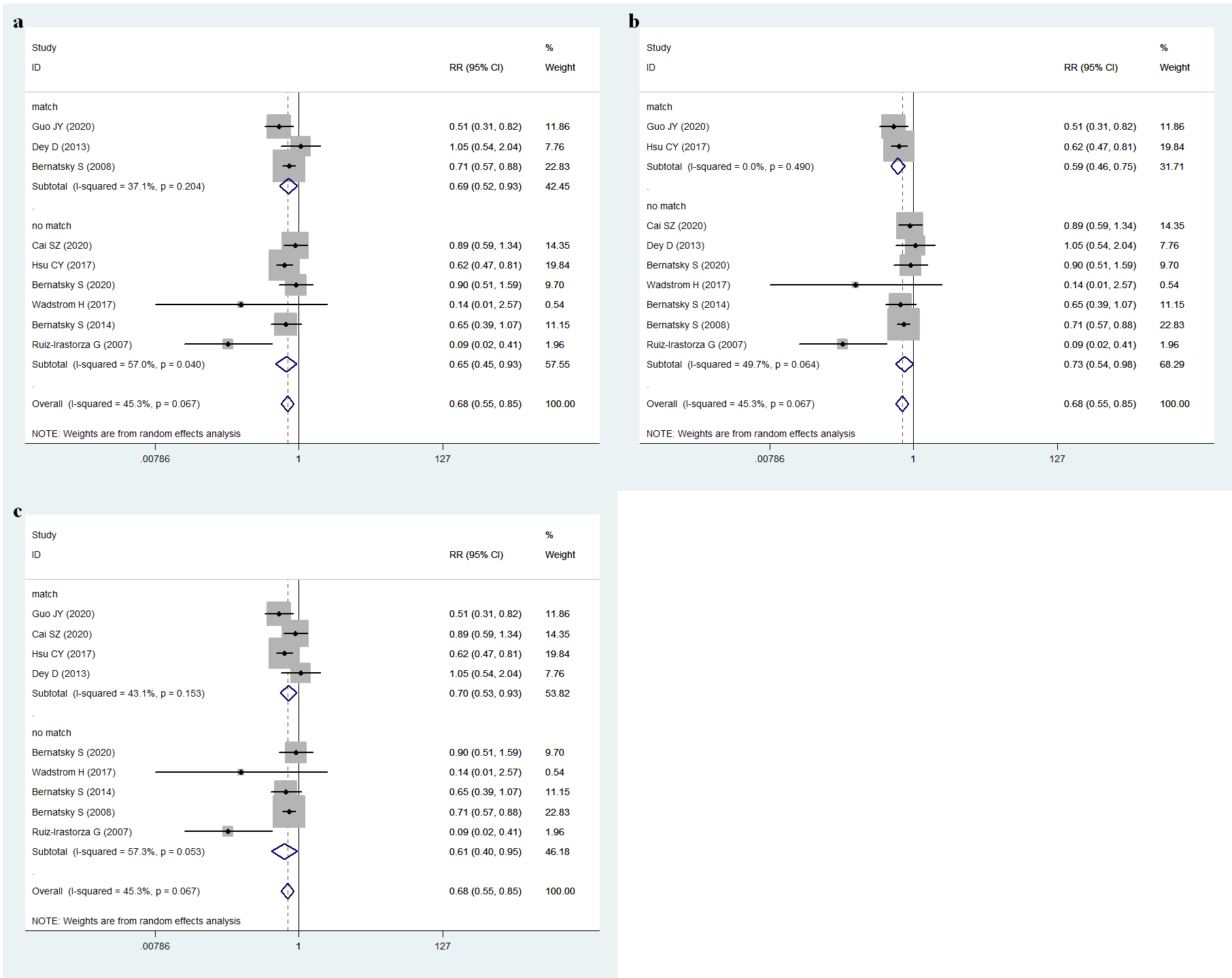

Supplement: Supplemental Material [file IANN_A_1981547_SM4237.zip › Supplemental files/Supplemental figure 1.tiff]

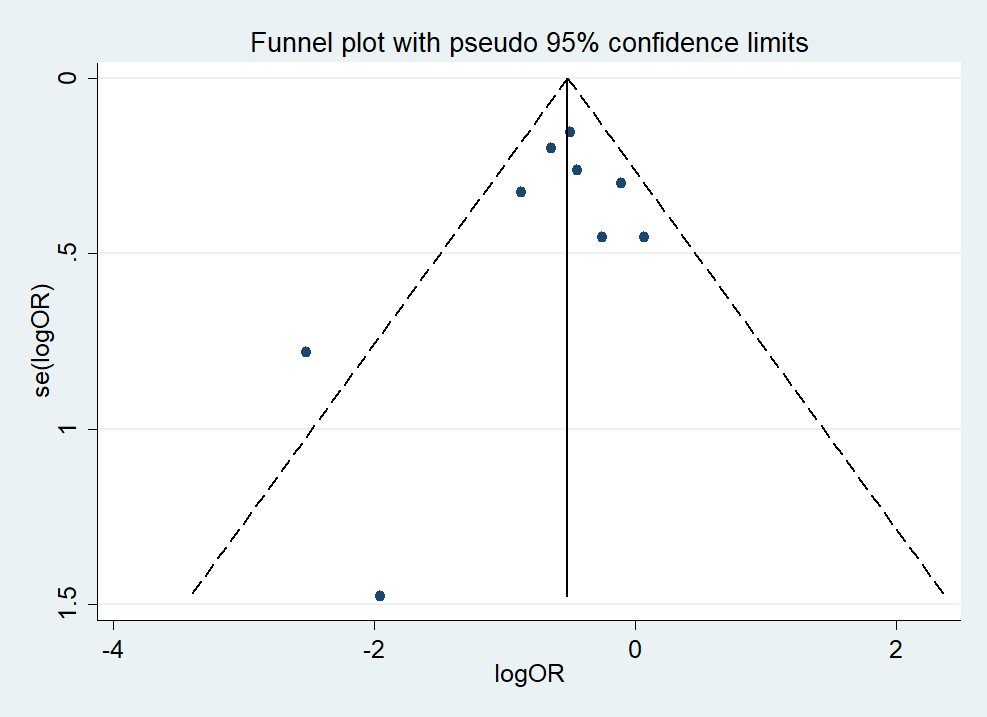

Supplement: Supplemental Material [file IANN_A_1981547_SM4237.zip › Supplemental files/Supplemental figure 2.tiff]

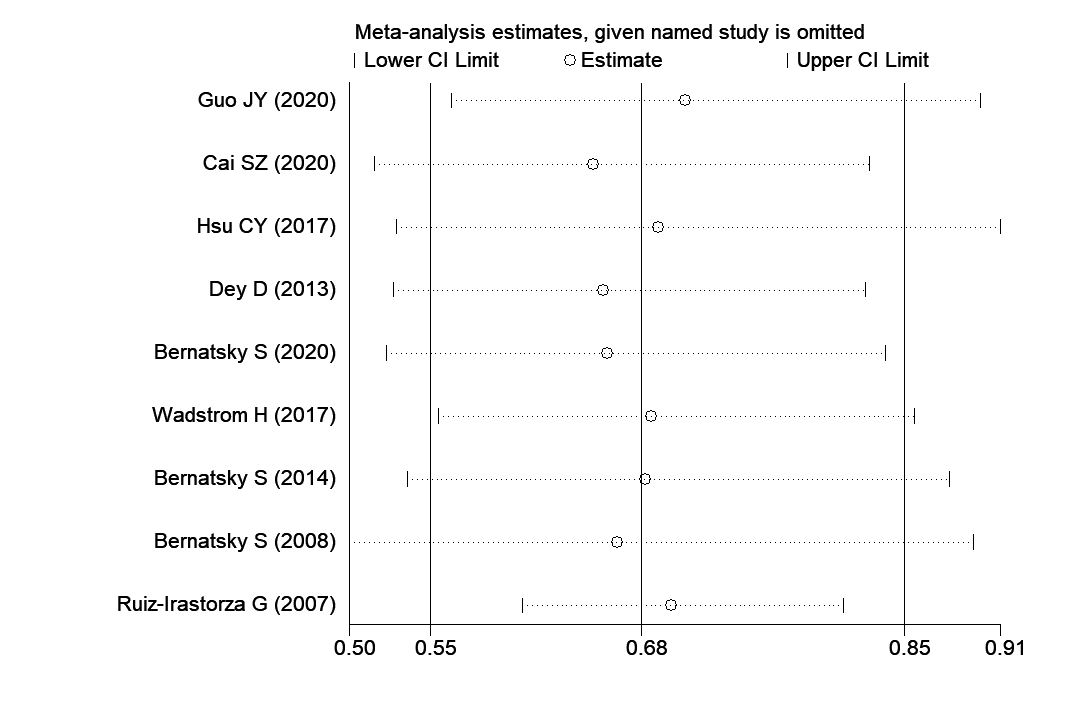

Supplement: Supplemental Material [file IANN_A_1981547_SM4237.zip › Supplemental files/Supplemental figure 3.tiff]
